# Clathrin-mediated endocytosis as an axis for the etiopathogenesis of Parkinson's disease

**DOI:** 10.1242/jcs.264368

**Published:** 2026-03-10

**Authors:** Amal Kaci, Susanne Herbst, Claudia Manzoni, Patrick A. Lewis

**Affiliations:** ^1^ The Royal Veterinary College, Royal College Street, Camden Town, NW1 0TU, UK; ^2^ UCL School of Pharmacy, Brunswick Square, London, WC1N 1AX, UK; ^3^ Queen Square UCL Institute of Neurology, Queen Square, London, WC1N 3BG, UK; ^4^ Aligning Science Across Parkinson's (ASAP) Collaborative Research Network, Chevy Chase, MD 20815, USA

**Keywords:** Clathrin-mediated endocytosis, Parkinson's disease, Parkinsonism, Synaptic transmission

## Abstract

Clathrin-mediated endocytosis (CME) is a core process in eukaryotic cells, providing a mechanism to selectively take up cargo from the extracellular space into the cytoplasm. Emerging data from both Mendelian and idiopathic forms of Parkinson's disease and parkinsonism syndromes supports a role for key players in CME in determining the risk of developing these neurodegenerative disorders. This Perspective summarizes the current genetic and functional evidence supporting a role for CME in Parkinson's disease, suggesting routes through which CME dysfunction could contribute to the etiopathogenesis of this disorder, and discussing the therapeutic potential for targeting CME in the context of neurodegeneration linked to Parkinson's disease and parkinsonism.

## Teaser

**Summary:** New insights into the genetics of Parkinson's disease reveal a potential role for clathrin-mediated endocytosis, opening up new avenues for investigating the pathobiology of Parkinson's disease and developing novel drugs.

## Introduction

**Fig. 1.**
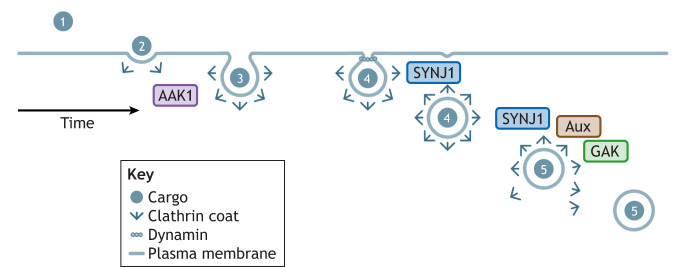
**Schematic of CME indicating points where genetic risk factors for Parkinson's disease intersect with this pathway.** The CME pathway comprises: (1) cargo recognition, (2) initiation and stabilization, (3) maturation, (4) fission, and (5) uncoating. AAK1 acts to coordinate the initiation of CME, whereas SYNJ1 is involved in the fission and uncoating stages, and GAK and auxilin (Aux) are required for uncoating of the clathrin-coated vesicles.

Clathrin-mediated endocytosis (CME) is a highly conserved eukaryotic system for the selective import of material into cells (Kaksonen and Roux, 2018). CME proceeds through several distinct stages – cargo recognition, initiation and stabilization of the clathrin vesicle, maturation, fission from the membrane and finally uncoating (Fig. 1). At the heart of this process is the invagination of the plasma membrane, and the formation of a geodesic clathrin cage around the nascent vesicle (Pearse, 1976; Anderson et al., 1978). This process is essential for cellular function across tissues and cell types, with specialized forms of CME functioning within the central nervous system in neuronal cell populations that play a crucial role in synaptic function (Maycox et al., 1992; Heuser, 1989). Disruption of this process has emerging implications in neurodegeneration. The past 10 years have witnessed the identification of multiple genetic links between the proteins and pathways regulating CME, and heightened risk of developing Parkinson's disease. Parkinson's disease is a neurodegenerative disorder characterized by a spectrum of symptoms including autonomic dysfunction (in particular constipation), prominent alterations in movement (notably tremor, rigidity and bradykinesia) and increased risk of cognitive dysfunction (Morris et al., 2024). It falls within a broader spectrum of parkinsonism syndromes all of which feature disrupted movement (Keener and Bordelon, 2016). Pathologically, Parkinson's disease is defined by the presence of Lewy bodies, intracellular aggregates of the protein α-synuclein, which helps regulate synaptic vesicle trafficking and neurotransmitter release. When α-synuclein abnormally accumulates within neurons, it contributes to neuronal dysfunction and death (Calabresi et al., 2023). Importantly, and perhaps directly relevant to the genetic links between Parkinson's disease and CME, there is increasing evidence supporting the cell-to-cell transfer of α-synuclein aggregates as a key contributing event in the pathogenesis of Parkinson's disease (Tofaris, 2022). In this Perspective, the contribution of four key regulatory proteins involved in CME and with robust genetic evidence linking them to Parkinson's disease – auxilin (encoded by *DNAJC6*), cyclin G-associated kinase (GAK), synaptojanin 1 (SYNJ1) and AP2-associated kinase 1 (AAK1) – to the etiology and pathogenesis of Parkinson's and parkinsonism will be discussed in the context of a potential cellular axis for the development of these disorders.

## The genetic evidence

Four core proteins involved in the central events of CME, auxilin, GAK, SYNJ1 and AAK1, have been directly linked to Parkinson's disease and parkinsonism. Loss-of-function mutations in DnaJ heat shock protein family (Hsp40) member C6 (*DNAJC6*) gene on chromosome 1, encoding auxilin, were identified in 2012 as the cause of autosomal recessive juvenile parkinsonism (Edvardson et al., 2012; Koroglu et al., 2013). Auxilin acts at the final stage of CME, binding to the engulfed clathrin-coated vesicle and recruiting the machinery required for clathrin uncoating (Fig. 1) (Ungewickell et al., 1995).

**Fig. 2.**
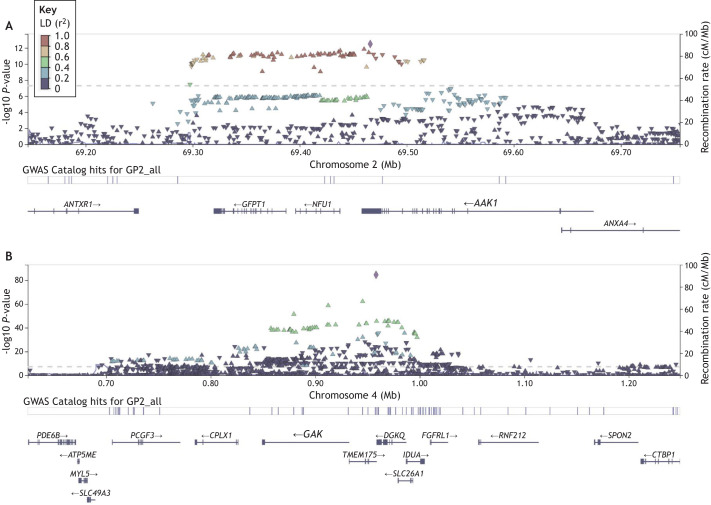
**The AAK1 and GAK loci associated with Parkinson's disease risk.** Data from GWASs indicating the −log10 *P*-value for significant association of individual single nucleotide polymorphisms (SNPs) with disease risk across each locus. The *y*-axis displays −log10 *P*-value; the *x*-axis displays chromosomal location on chromosome 2 (A) and chromosome 4 (B), along with annotated genes at each locus. Each triangle represents an individual SNP, with purple diamonds in each image the lead risk SNP at the locus. Color coding for SNPs relates to the level of linkage disequilibrium, indicating the co-inheritance of allelic blocks due to reduced recombination (see key). Locus plots were accessed through the Parkinson's genome browser (https://pdgenetics.shinyapps.io/GWASBrowser; Grenn et al., 2020).

GAK is another CME protein associated with Parkinson's disease. *GAK* has emerged as a candidate gene for Parkinson's disease risk from multiple genome-wide association studies (GWASs) (Hamza et al., 2010; Pankratz et al., 2009; Rhodes et al., 2011). Variants at the *GAK* locus are associated with significantly increased lifetime risk for idiopathic Parkinson's disease, although *GAK* is one of two candidate genes for being the functional driver of risk at this locus [the other being transmembrane protein 175 (*TMEM175*), a lysosomal ion channel] (Jinn et al., 2019; Nagle et al., 2016) (Fig. 2). *GAK* is located on chromosome 4 and shares substantial sequence conservation with auxilin at its C-terminus and contains an additional serine/threonine kinase domain at the N-terminus. Similar to auxilin, GAK is involved in uncoating of the clathrin vesicle (Fig. 1) (Greener et al., 2000).

Variants in a third protein involved in CME, SYNJ1, cause a complicated early-onset disorder encompassing symptoms including generalized seizures and progressive parkinsonism (Krebs et al., 2013; Quadri et al., 2013). SYNJ1, encoded by the *SYNJ1* gene on chromosome 21 is a lipid phosphatase involved in the dephosphorylation of a number of lipids moieties and plays a key role in the uncoating of clathrin-coated vesicles (Fig. 1) (Choudhry et al., 2021).

**Fig. 3.**
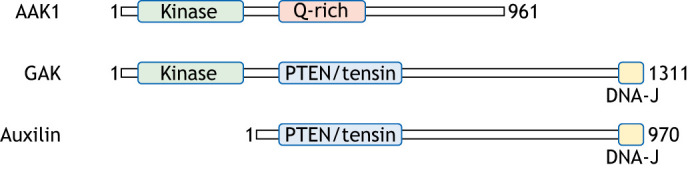
**Schematic representations of AAK1, GAK and auxilin, indicating domain conservation between and across these three proteins.** Note the conservation of the kinase domains between AAK1 and GAK (both members of the Numb family of kinases), and of the C-terminals of GAK and auxilin, where the DNA-J domain is crucial for maintaining interactions with cytosolic chaperone proteins.

A fourth CME regulator, AAK1, has been nominated as a Parkinson's disease risk gene in the most recent meta-analysis of Parkinson's GWASs (Fig. 2) (Leonard and Global Parkinson's Genetics Program, 2025, preprint), having previously been implicated (but not reaching genome-wide significance) in a smaller scale GWAS examining age-at-onset in Parkinson's (Latourelle et al., 2009). *AAK1* is located on chromosome 2 and encodes a kinase that coordinates the initiation and maturation of clathrin-coated vesicles at the cell surface via the phosphorylation of clathrin adaptor proteins (Fig. 1) (Conner and Schmid, 2002). Intriguingly, AAK1 shares homology with the kinase domain of GAK, with both belonging to the Numb associated kinase family (four kinases with conserved roles in endocytic pathways), linking AAK1, GAK and auxilin mechanistically (Fig. 3), with the two kinases bookending the process of CME (Huang et al., 2023).

## The functional data

GAK has been the subject of a number of functional studies investigating their potential links to neurodegeneration and Parkinson's disease. In *Drosophila melanogaster*, loss of the GAK ortholog (somewhat confusingly called Auxilin in the fly) results in locomotor deficits (Song et al., 2017). Further investigations in *Drosophila* have highlighted a potential role for GAK in the autophagy-lysosomal system, in addition to its canonical function in CME (Zhang et al., 2025, 2023). These findings open up the possibility that GAK contributes to the regulation of catabolic pathways in the cell, processes with strongly established functional links to Parkinson's disease (Manzoni and Lewis, 2013). Supporting this, analyses of human brain tissue and cellular models suggest that GAK can modify the expression of *SNCA* (encoding α-synuclein) and modulate its toxicity – although is not clear whether this impact derives from the influence of GAK on CME or upon the autophagy-lysosomal pathway (Dumitriu et al., 2011). Intriguingly, GAK has also been also identified in an unbiased proteomic screen as an interactor of leucine-rich repeat kinase 2 (LRRK2), linking GAK to one of the most important sources of genetic risk for Parkinson's disease (coding variants in *LRRK2* are a common cause of the disorder) (Beilina et al., 2014). LRRK2 is deeply implicated in the regulation of lysosomal damage responses and synaptic vesicle trafficking, and a potential regulatory connection between LRRK2 and GAK could represent a common pathway to disease. Further *Drosophila* studies support an interplay between LRRK2, GAK and α-synuclein, with *aux* and *Lrrk* loci modifying α-synuclein aggregation phenotypes in the fly (Olsen and Feany, 2021).

Despite their mechanistic similarity, auxilin and GAK exhibit distinct phenotypes in knockout mouse models. Knockout of auxilin results in disruption of CME in the central nervous system, leading to specific synaptic and dopaminergic deficits (Cheng et al., 2023; Vidyadhara et al., 2023; Yim et al., 2010). In contrast, knockout of GAK – ubiquitously expressed in mammalian tissues, unlike the predominantly CNS-expressed auxilin – is lethal in mice (Lee et al., 2008). The divergence in phenotype severity between GAK and auxilin loss of function might explain why pathogenic variants of *DNAJC6* are Mendelian in nature, whereas only subtle variation at the *GAK* locus has been linked to increased risk of Parkinson's disease. Notably, disease-associated loss-of-function Mendelian variants in *GAK* have not been identified in humans to date, likely reflecting that homozygous loss-of-function GAK variants would be lethal *in utero* or during the perinatal period, as observed in mice.

The role of auxilin has also been examined in human cellular models. Loss-of-function mutations result in synaptic and developmental defects in induced pluripotent stem cells (iPSC)-derived dopaminergic neurons and brain organoids obtained from individuals with *DNAJC6*-linked parkinsonism (Abela et al., 2024; Wulansari et al., 2021). These studies also highlight the potential tractability of pathogenic variants in *DNAJC6* to gene therapy, although the wider applicability of such interventions beyond individuals carrying mutations in the gene remains unclear. Additional evidence for interplay between auxilin and GAK comes from biofluids and cells derived from individuals carrying pathogenic *DNAJC6* variants, which display increased GAK levels in cerebrospinal fluid (Ng et al., 2020). Auxilin also appears to be directly linked to LRRK2; iPSC neurons derived from individuals with pathogenic *LRRK2* variants show increased phosphorylation of auxilin (Nguyen and Krainc, 2018).

Loss or mutation of *Synj1* has a substantial impact on CME in mouse and cellular models (Cao et al., 2017), and exhibits phenotypic overlap with auxilin, suggesting functional convergence (Ng et al., 2023b).

In contrast to GAK, SYNJ1 and auxilin, there is a paucity of studies directly examining mechanisms that might link AAK1 to Parkinson's disease. Although CME has been studied extensively, and by implication the function of AAK1 in regulating vesicle initiation, direct experimental modulation of AAK1 in Parkinson's disease models remains limited. Thus, although AAK1 is genetically implicated in Parkinson's disease risk, its functional role remains comparatively understudied.

## CME as an axis for Parkinson's disease risk

As noted above, CME has been highlighted by multiple genetic and functional studies as being linked to Parkinson's disease. In addition to the canonical roles of AAK1, GAK, SYNJ1 and auxilin in regulating CME (and their genetic associations with Parkinson's disease), α-synuclein, the major constituent of Lewy bodies (the intracellular inclusions found in the brains of people with Parkinson's disease) and a key genetic contributor to disease, has also been connected to several stages of CME.

α-Synuclein has been reported to facilitate endocytosis in human cell models through its localization with the clathrin adaptor AP2, thereby regulating phosphatidylinositol 4,5-bisphosphate levels (Schechter et al., 2020), an interaction that has recently been replicated (Vargas et al., 2025). α-Synuclein has also been reported to interact directly with clathrin latices (Vargas et al., 2023) and to regulate the clathrin-dependent localization of the dopamine transporter (Kisos et al., 2014). Intriguingly, α-synuclein has also been reported to act through SYNJ1 (Song et al., 2023), with a possible further connection to the uncoating machinery via Hsc70 (Banks et al., 2020).

As previously noted, LRRK2 has multiple connections to CME through its interactions with auxilin and GAK. Beyond these interactions, LRRK2 activity has been linked to AP2 (Heaton et al., 2020; Liu et al., 2021) and to endophilin A1 (Bademosi et al., 2023), another important component of the regulatory apparatus surrounding CME (Milosevic et al., 2011). In each case, there is evidence for phospho-regulation of these proteins, with LRRK2 phosphorylating specific residues in these proteins and potentially regulating their activity. Collectively, these findings support the existence of a tightly integrated signaling network connecting Parkinson's risk genes to the regulation of CME. Intriguingly, mutations in AP2 have recently been reported to cause hereditary spastic paraplegia, a movement disorder distinct from Parkinson's disease but with partially overlapping etiology (Diarra et al., 2024).

**Fig. 4.**
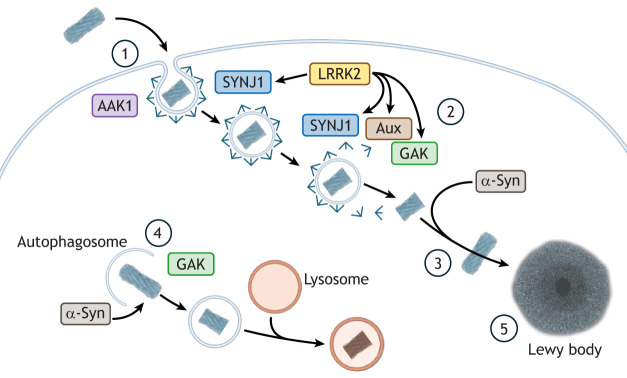
**A putative model for the role played by CME in α-synuclein aggregate propagation in Parkinson's disease.** (1) Aggregates formed of α-synuclein are engulfed via CME, with variation in AAK1 altering the efficiency of this process. (2) Uncoating of clathrin-coated vesicles is altered by the activity of GAK, auxilin (Aux) and SYNJ1, proteins whose activity that has been reported to be regulated by LRRK2. (3) These changes alter the potential for α-synuclein to form self-propagating foci within the cell. (4) In parallel, GAK-mediated modulation of lysosomal aggregation might contribute to altered catabolism of α-synuclein (α-Syn). (5) Together, these processes increase proteotoxic load in neurons, ultimately contributing to neurodegeneration.

The emerging genetic evidence directly implicating the regulatory machinery governing CME in the etiology and pathogenesis of Parkinson's disease and parkinsonism, and in particular the convergence on proteins with shared or overlapping functions, supports a common mechanism of disease. Several aspects of CME dysfunction could contribute to the loss of dopaminergic neurons in Parkinson's disease and parkinsonism. One potential mechanism is through modulation of protein aggregate uptake and disposal (Hijaz and Volpicelli-Daley, 2020). In this scenario, genetic variants impacting on CME function might modulate the efficiency of α-synuclein aggregate propagation from cell to cell, with a potential secondary effect upon degradative pathways linked to lysosomal mediated by GAK. This could drive a progressive spread of dysfunction through the brain, ultimately resulting in neuronal death (Fig. 4).

Beyond aggregate trafficking, CME genetic variants impact specific aspects of synaptic function. Disruption or dysfunction of synaptic vesicle recycling has been implicated as a disease mechanism in Parkinson's disease (Ng and Cao, 2024). Synaptic frailty linked to CME has previously been suggested as contributor to the disease process in Alzheimer's disease (Yao, 2004), highlighting mechanistic overlap with other neurodegenerative disorders. Notably, these possibilities are not mutually exclusive; indeed, it is entirely plausible that genetically driven alterations in CME act through multiple parallel pathways – modulating synaptic pathology and impacting on neuronal catabolic capacity.

## Targeting CME in Parkinson's disease

One key implication of the increasing evidence connecting CME to Parkinson's disease and parkinsonism is the potential for key components of this pathway to serve as targets for drug development. Taking the core machinery discussed above, two candidates stand out as tractable targets – AAK1 and GAK. As kinases, they benefit from a deep and extensive body of pharmaceutic and pharmacological research into developing kinase inhibitors from both industry and academic laboratories (Ferguson and Gray, 2018). Specific and potent experimental inhibitors have been developed for AAK1 and are commercially available (Kostich et al., 2016; Wells et al., 2020), and this is also the case for GAK (Kovackova et al., 2015). Whether inhibition of these kinases is beneficial in the context of Parkinson's disease, however, remains an open question. A granular understanding of whether reducing or increasing their activity is therapeutical desirable still needs to be established. Given the interplay and overlap with LRRK2 biology, it is possible that part of the consequence of inhibiting LRRK2 activity could involve alterations in CME – noting that LRRK2 is the subject of ongoing advanced clinical trials with both kinase inhibitors and antisense oligonucleotides (Kluss et al., 2022). Gaining a deeper comprehension of the signaling events around LRRK2, the effects of inhibiting or modulating its activity, and how these impact CME could open new routes for targeting CME in Parkinson's disease.

Beyond enzymatic targets, there are increasing opportunities with novel modalities to modulate gene and protein function in the central nervous system. *DNAJC6*, where homozygous loss of function results in disease, exemplifies such an opportunity, with ongoing efforts to restore function through gene therapy (Abela et al., 2024), following on from previous efforts to develop treatments for mutations in the dopamine transporter gene (Ng et al., 2021 2023a). As our comprehension of the overlap between Parkinson's and CME increases, new ways of targeting genes and proteins using antibody or gene therapy approaches will provide new opportunities to modulate this pathway (McFarthing et al., 2024).

## Caveats, confounds and complications

Several important caveats and confounding factors complicate interpretation of the convergence of genetic disease risk on CME in Parkinson's disease and parkinsonism. First, there are challenges in understanding the relationship between Parkinson's disease and parkinsonism, which include diseases caused by pathogenic variants in *DNAJC6* and *SYNJ1*. The clinical presentations of the latter, coupled to uncertainty related to the neuropathological characteristics (which remain poorly defined), indicate that there are important distinctions between these disease processes and Parkinson's disease as a clinicopathological entity. Although this does not exclude the possibility of common pathways, especially given the shared aspects of movement disorder dysfunction, it does suggest that caution should be taken when extrapolating findings from these disorders to idiopathic Parkinson's disease.

Regarding the associations of *GAK* and *AAK1* with increased risk of idiopathic Parkinson's disease, there is a level of uncertainty intrinsic and unavoidable in interpreting GWASs (Mountjoy et al., 2021). At both loci, several candidate genes are present. The chromosome 4 risk locus that includes *GAK* also includes *TMEM175*, which has strong functional evidence linking it to Parkinson's disease (Hu et al., 2022; Wu et al., 2023).

Similarly, *AAK1* is one of three candidates at the chromosome 2 locus (*NFU1* and *GFPT1* being the other genes; see Fig. 2), and at present there are insufficient functional data to confidently identify the most likely driver of the association. Furthermore, the direction and consequences of transcript-level variation at these loci have not yet been fully characterized – information that will be crucial for interpreting and understanding the nature of any genetic risk involving *AAK1* and *GAK*.

A further concern arises when considering the therapeutic targeting of CME – CME is essential for cellular function across all tissues and cell types. Although the evidence recounted above supports a specific deficit in a subset of central nervous system cells leading to neurodegeneration, selectively targeting this deficit without causing on-target effects in other tissues represents a major challenge.

## Conclusions and future directions

There is a growing body of evidence linking CME to the events that lead to neurodegeneration in Parkinson's disease and parkinsonism. In the absence of disease-modifying therapies for these disorders, expanding functional data on the role of CME in Parkinson's disease should be a research priority. At the same time, genetic and functional analyses of disease mechanisms related to CME offer excellent potential to yield new and provocative insights into the nature and biology of this pathway.

Several areas relevant to the role of CME in Parkinson's disease warrant further investigation. First, uncertainty remains regarding the identity of the causative genes at the *AAK1* and *GAK* loci. Further functional analysis of these loci, with a particular focus on gene expression and expression quantitative trait locus analysis, will provide further insights into this.

There is also a clear need for further investigation of the putative roles of these proteins in Parkinson's disease. Cellular and animal models provide opportunities to test how closely their activities and functions overlap with established Parkinson's disease pathways, as well as their influence on cellular and organismal phenotypes relevant to disease. As noted above, a deeper understanding of the impact of CME on aggregate propagation and synaptic function in disease would be especially valuable. There is the intriguing possibility that these two aspects of Parkinson's pathobiology might converge through CME, with synaptic dysfunction coupled to trans-synaptic propagation or spread of protein aggregates (as recently described for tau aggregates in progressive supranuclear palsy, a movement disorder related to Parkinson's disease) (McGeachan et al., 2025). With increasingly sophisticated models and tools available to investigate these aspects, and with the ability to modulate the activities of AAK1 and GAK in particular, it will soon be possible to test whether CME plays a key role in these aspects of Parkinson's disease biology.

Taken together, genetic and mechanistic findings position CME at key juncture for Parkinson's disease pathobiology.
